# Retrieval-Dependent Mechanisms Affecting Emotional Memory Persistence: Reconsolidation, Extinction, and the Space in Between

**DOI:** 10.3389/fnbeh.2020.574358

**Published:** 2020-09-29

**Authors:** Zuzana Vaverková, Amy L. Milton, Emiliano Merlo

**Affiliations:** ^1^School of Psychology, University of Sussex, Brighton, United Kingdom; ^2^Department of Psychology, Behavioural and Clinical Neuroscience Institute, University of Cambridge, Cambridge, United Kingdom

**Keywords:** associative memory, reconsolidation, extinction, limbo, prediction error, memory persistence

## Abstract

Maladaptive emotional memories contribute to the persistence of many mental health disorders, and therefore the prospect of disrupting these memories to produce long-term reductions in relapse is of great clinical appeal. Reducing the impact of maladaptive emotional memories on behaviour could be achieved by two retrieval-dependent manipulations that engage separate mnemonic processes: “reconsolidation disruption” and “extinction enhancement.” Extinction occurs during a prolonged re-exposure session in the absence of the expected emotional outcome and is widely accepted as reflecting the formation of a new, inhibitory memory that prevents behavioural expression of the original trace. Reconsolidation, by contrast, involves the destabilisation of the original memory, allowing for subsequent updating and restabilisation in specific brain regions, unless the re-stabilization process is prevented through specific pharmacological or behavioural interventions. Both destabilisation of the original memory and memory extinction require that re-exposure induces prediction error—a mismatch between what is expected and what actually occurs—but the parameters that allow reconsolidation and extinction to occur, and control the transition between them, have not been well-characterised. Here, we review what is known about the induction of memory destabilisation and extinction, and the transition period that separates these mnemonic processes, drawing on preclinical and clinical examples. A deeper understanding of the processes that determine the alternative routes to memory persistence or inhibition is critical for designing new and more reliable clinical treatments targeting maladaptive emotional memories.

## Introduction

To survive and reproduce, animals need to learn about the motivational significance of environmental cues; predicting the presence of a predator or a potential mate based on learned or conditioned stimuli (CSs) in the environment allows animals to prepare a behavioural response rather than simply reacting to an unpredicted threat or reward, acting as an unconditioned stimulus (US). The capacity to form pavlovian associations between CSs and USs, therefore, confers a clear survival advantage. However, under certain conditions, this adaptive learning system can produce memories that are overly strong and dominant over behavior. These persistent, *maladaptive* memories are thought to underlie some of the most prevalent psychiatric conditions including post-traumatic stress disorder, specific phobia, substance dependence, and binge eating disorder. It is hypothesised (Everitt et al., 2001; Hyman and Malenka, 2001; Milton and Everitt, 2010; Torregrossa et al., 2011) that such disorders are established and maintained through aberrant learning processes that hijack the neural mechanisms necessary for the persistence of emotional memories. Maladaptive memories are pathologically persistent and greatly increase the risk of relapse even after successful treatment (Parsons and Ressler, 2013). Therefore, disruption or suppression of maladaptive memories may potentially offer an innovative form of treatment to overcome both fear and substance abuse disorders (Singewald et al., 2015; Everitt et al., 2018; Monfils and Holmes, 2018).

Reducing the impact of maladaptive memories on behavior could be achieved by targeting one of two memory retrieval-dependent processes that engage different mnemonic processes: disrupting memory reconsolidation or enhancing extinction. Both of these processes depend upon re-exposure to the pavlovian CSs or “trigger stimuli,” and on inducing a “mismatch” between what is expected and what occurs (more formally referred to as “prediction error”). Furthermore, although induced by re-exposure, the relationship between memory retrieval, memory reconsolidation, and extinction is non-linear and governed by “boundary conditions” that depend upon both the age and the strength of the original memory (Suzuki et al., 2004; Alberini et al., 2006). In a laboratory setting, it is possible to control memory age and strength and consequently boundary conditions, but this is not possible in the clinical situation. We argue that currently, one of the greatest challenges in translating preclinical memory modification research to the clinical situation is knowing *when* each of the different mnemonic processes has been engaged. Here, we review what is known of the molecular mechanisms underlying the destabilisation of consolidated memories in reconsolidation, and how these partially overlap with those engaged when a new extinction memory is formed. Although maladaptive memories can be pavlovian or instrumental, here our focus is on pavlovian memories as these have been most extensively studied across the appetitive and aversive domains.

## Re-Exposure to Pavlovian CSs Induces Different Mnemonic Processes Depending on The Extent of Re-Exposure

**Figure 1:**
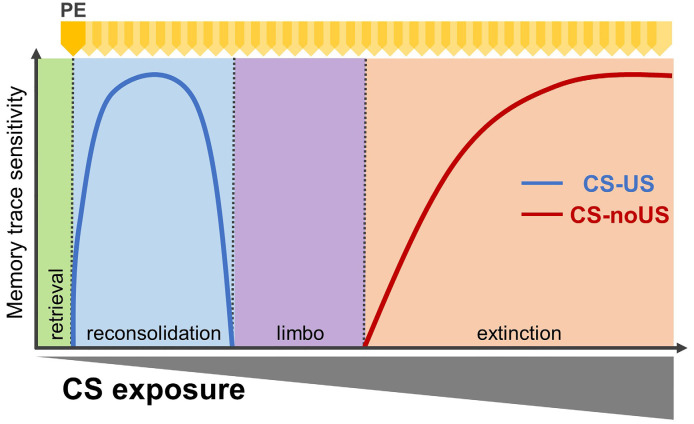
Alternative retrieval-dependent memory processes affecting pavlovian associative memories. Fully consolidated associative memories engage four alternative retrieval-dependent processes, presented in boxes: retrieval alone (green), reconsolidation (destabilisation /re-stabilisation; blue), limbo (purple), or extinction (orange). Prediction error, as the discrepancy between what is predicted by the conditioned stimuli(CS)–unconditioned stimulus (US) memory and what occurs, is necessary for all but retrieval alone. Lines indicate which memory process is dominant depending on CS re-exposure (number of events or duration). Dominant memory traces are those sensitive to amnestic interventions. With a limited number of CS re-exposures, amnestic manipulations reduce subsequent expression of the conditioned response (CR) and are interpreted as engaging memory reconsolidation. By contrast, extended CS re-exposure leads to the formation of extinction memory, and amnestic treatments will prevent this new memory from forming. For intermediate CS re-exposures, neither reconsolidation nor extinction is engaged, and the memory trace becomes insensitive to amnestic agents. PE: prediction error (yellow arrowheads).

Following the acquisition of a pavlovian memory, re-exposure to the CS alone can induce one of four memory processes: retrieval, reconsolidation, “limbo” or extinction (Figure 1; Eisenberg et al., 2003; Pedreira and Maldonado, 2003; Merlo et al., 2014, 2018). A brief presentation of the CS transiently returns the associative memory to a labile state, sensitive to disruption, followed by a re-stabilization process (Nader et al., 2000). By contrast, prolonged CS re-exposure produces inhibition of the conditioned response (CR) through extinction. It is hypothesised that behavioural inhibition results from the formation of a new “CS-noUS” associative memory that prevents expression of the original CS-US memory without erasing it (Bouton, 2004).

**Figure 2:**
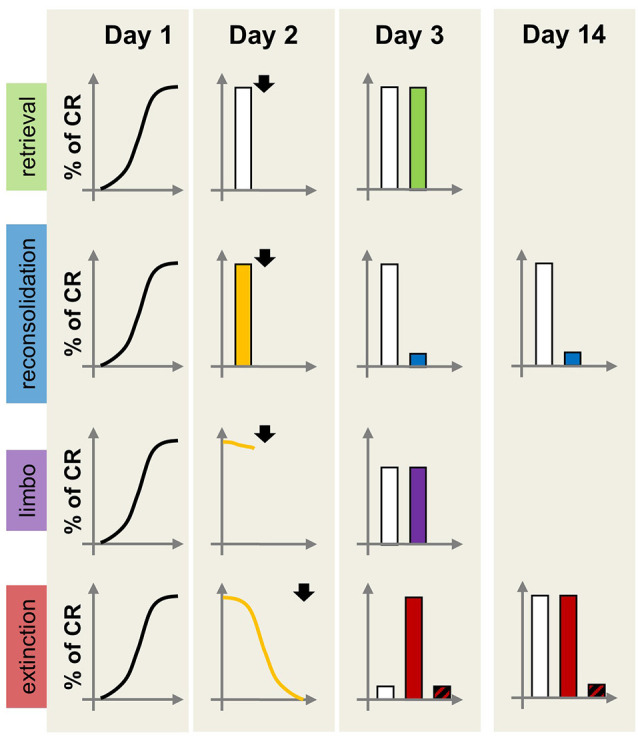
Schematic representation of the effects of amnestic or hypermnestic manipulations on memory persistence as a function of the dominant memory process. On Day 1, all individuals acquire the same pavlovian CR. On Day 2, different groups receive different (increasing) numbers of CS re-exposure producing four experimental conditions: retrieval only (brief CS exposure without prediction error, top row), reconsolidation (brief CS exposure with prediction, second row), limbo (intermediate CS exposure, third row) or extinction (prolonged CS exposure, bottom row). Immediately after each CS re-exposure condition, individuals receive an amnestic or control treatment (e.g., protein synthesis inhibitor; arrowhead). For the extinction condition, the plot also shows the effect of a hypermnestic drug (e.g., D-cycloserine). On Day 3 long-term memory is tested, with outcomes differing depending on the experimental condition. Retrieval condition: the amnestic treatment (green bar) does not affect the CR, indicating that the CS-US memory did not destabilize on Day 2. Reconsolidation condition: the amnestic treatment (blue bar) produces memory disruption, due to the interference of the drug with the CS-US memory re-stabilization process at Day 2. Limbo condition: there is no difference in CR between vehicle- and drug-treated groups (purple bar), indicating that the original CS-US memory was not destabilized at Day 2, and no engagement of extinction mechanisms. Extinction condition: the amnestic-treated group (red bar) shows high CR, consistent with extinction disruption during Day 2; it also shows extinction in the group receiving the hypermnestic drug. On Day 14 the second row shows maintenance of the low CR in animals receiving the amnestic treatment after memory reactivation. By Day 14, the vehicle-treated extinction group shows the spontaneous recovery of the CR, whereas the hypermnestic-treated group (diagonally striped black and red bar) maintains a low CR due to enhanced extinction.

Both reconsolidation disruption and extinction enhancement are potentially valuable treatment strategies able to reduce the impact of cue-dependent maladaptive memories on behavior (Figure 2). Research on extinction profoundly influenced the development of prolonged exposure therapy for anxiety disorders and continues to inform refinements to this therapeutic approach (Craske et al., 2014). However, it is worth noting that prolonged exposure is not effective for all patients, with relapse and re-emergence of the maladaptive memory being a relatively common occurrence (Holmes et al., 2014).

**Figure 3:**
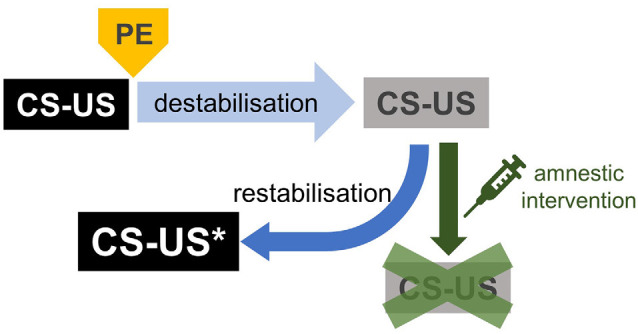
CS-US memory processing after a brief retrieval. In the presence of prediction error (PE), brief retrieval induces CS-US memory destabilisation. Destabilised or labile CS-US memories are again susceptible to amnestic interventions, as they were during the consolidation phase. Labile CS-US memories are restabilised by a neural process dependent on *de novo* gene expression and protein synthesis. Restabilised CS-US memories return to a stable state, impervious to amnestic agents. Notably, reconsolidated CS-US memories become more precise and longer-lasting (CS-US*, see text).

Reconsolidation-based interventions are being actively investigated as an alternative therapeutic approach for patients who are not responsive to prolonged exposure therapy. A considerable number of studies have investigated the effects of pharmacological interventions aimed at disrupting memory reconsolidation, mostly in animal analogs of mental health disorders, though some small-scale patient studies have also been conducted. Reconsolidation consists of the two dissociable processes of memory *destabilisation* and *restabilisation* (Figure 3; Ben Mamou et al., 2006; Milton et al., 2013; Ferrer Monti et al., 2016). “Destabilisation” refers to the hypothetical process initiated by memory retrieval in presence of prediction error, by which the original CS-US memory becomes once again modifiable and regains sensitivity to amnestic treatments. To return to a stable state, the destabilised CS-US memory undergoes a re-stabilization process supported by, among other mechanisms, *de novo* gene expression, and protein synthesis. Much of the literature to date has focused on the re-stabilization process and identifying drugs that block this process to provide potential amnestic agents for use in humans. A large number of pharmacological targets have been identified, with a particular focus on drugs that are readily translatable to humans. These include the β-adrenergic receptor antagonist propranolol (Debiec and Ledoux, 2004; Diergaarde et al., 2006; Brunet et al., 2008; Milton et al., 2008; Kindt et al., 2009; Soeter and Kindt, 2010) and the glucocorticoid antagonist mifepristone (Jin et al., 2007; Taubenfeld et al., 2009; Pitman et al., 2011). However, this compelling evidence is challenged by several studies showing only limited or no effects of propranolol on aversive (Muravieva and Alberini, 2010; Wood et al., 2015; Schroyens et al., 2017) and appetitive memory reconsolidation (Milton et al., 2012; Pachas et al., 2015; Dunbar and Taylor, 2016). One potential account of these apparent discrepancies is that memory destabilisation was not engaged in those studies in which propranolol was not effective—namely, that these studies had not overcome the “boundary conditions” of reconsolidation. This explanation is supported by a series of case studies in which propranolol was effective when administered before memory reactivation when the expectations of PTSD patients had been violated, but ineffective when their expectations had not been violated (Kindt and van Emmerik, 2016). Thus, we would argue that one of the greatest barriers to the translation of reconsolidation-based interventions to the clinical situation is the reliable engagement of the memory destabilisation process. Furthermore, a better understanding of some of the subtleties in boundary conditions—along with a non-behavioural, independent marker of memory destabilisation—would likely provide great insight into the apparent fragility of reconsolidation interference effects.

## Destabilising The Original Memory

### Boundary Conditions

Studies in both vertebrates and invertebrates have shown that destabilisation of the original CS-US memory at retrieval relies upon a specific amount of prediction error—enough for the original memory to be sufficiently inaccurate to require updating, but not so much that the experience is consolidated as a new memory (see Osan et al., 2011; Gershman et al., 2017 for computational perspectives on boundary conditions). Prediction error, the discrepancy between the expected and actual outcome following a CS presentation, is necessary to destabilise both appetitive and aversive memories in crabs (Kaczer et al., 2011; López et al., 2016), rats (Morris et al., 2006; Reichelt et al., 2013), and humans (Sevenster et al., 2013; Das et al., 2015). Typically, for pavlovian CSs, a brief duration of re-exposure or a small number of CS re-exposures is sufficient to induce memory destabilisation without engaging extinction, for both appetitive and aversive memories and across species (Eisenberg et al., 2003; Pedreira and Maldonado, 2003; Flavell and Lee, 2013).

Relatively few studies have undertaken a parametric investigation of the conditions affecting memory destabilisation. More frequently, boundary conditions have been discovered when a memory reactivation session fails to induce sufficient prediction error to destabilise the memory. Predictions of specific boundary conditions are rare (though see Osan et al., 2011; Gershman et al., 2017 for predictions based on computational accounts) but two boundary conditions that have been observed across multiple studies are the strength and age of the original memory. Importantly, however, these boundary conditions do not mean that the memory *cannot* be destabilised, only that it cannot be destabilised under the same conditions as weaker or more recent memories. For example, a strong contextual fear memory required 10 min of context re-exposure to induce destabilisation, as compared to 3 min for a contextual fear memory trained under “standard” conditions (Suzuki et al., 2004), while the same reactivation procedure led to extinction for a weakly-trained appetitive pavlovian memory and reconsolidation for a strongly-trained pavlovian memory (Reichelt and Lee, 2013).

Similarly, acquisition-to-retrieval intervals modify the requirements for memory destabilisation. Early observations suggested that memories older than 14 days did not destabilise (Milekic and Alberini, 2002). However, older associative memories destabilise if the CS re-exposure session is extended (Suzuki et al., 2004), and it is possible to destabilise strongly trained pavlovian CS-cocaine memories even after many weeks (Lee et al., 2006b). Furthermore, in small-scale clinical trials, both strong and old memories have been shown to destabilise under specific reactivation conditions. The maladaptive memories underlying spider phobia were destabilised and disrupted by the administration of the beta-blocker propranolol when patients believed that they would need to pick up a tarantula and were stopped just before doing so (Soeter and Kindt, 2015). Similarly, PTSD patients showed a steep decline of fear symptoms after receiving a reconsolidation-based intervention during which patients were required to rate the extent to which the expectations of their response to trauma re-exposure had been violated (Kindt and van Emmerik, 2016; Kessler et al., 2018).

There is, however, the potential for unfalsifiability when considering memory destabilisation and boundary conditions. When a reactivation session induces a memory to become once again susceptible to amnestic agents, it is considered that the parameters of that reactivation session have induced memory destabilisation. If the reactivation session does not induce the memory to become susceptible to anamnestic agent, then boundary conditions are invoked. Independent neural markers of memory destabilisation, that do not rely only on behavior, would therefore be a valuable addition to studies of memory destabilisation.

### Neural Markers of Memory Destabilisation

Several neurochemical and molecular mechanisms contributing to memory destabilisation have been identified. At the cell surface level, destabilisation depends upon specific subtypes of glutamatergic receptors, dopamine receptors, and L-type voltage-gated calcium channels.

One of the first studies of the neurochemical mechanisms of memory destabilisation showed that the GluN2B-subtype-selective NMDA receptor (NMDAR) antagonist ifenprodil could block the destabilisation of auditory fear memory (Ben Mamou et al., 2006, and subsequent replication by Milton et al., 2013). Furthermore, reducing the relative number of GluN2B-containing NMDARs in a mouse selectively overexpressing GluN2A-containing NMDARs in BLA projection neurons at the time of memory reactivation, led to fear memories that did not destabilise when those of controls undergoing the same memory reactivation procedure did (Holehonnur et al., 2016). This supports hypotheses asserting that the balance between GluN2A-NMDAR and GluN2B-NMDAR expression may mediate resistance to memory destabilisation (Zhang et al., 2018).

Although AMPAR antagonism has been shown to prevent memory retrieval without impairing memory destabilisation (Milton et al., 2013), it appears that as for NMDARs, specific types of AMPARs may be necessary for memory destabilisation. There is a transient reduction in AMPARs expressing the GluA2 subunit (which makes the receptor calcium-impermeable) at the neuronal membrane following fear memory reactivation, which is followed by an increase in AMPAR expression within 7 h of the reactivation session (Rao-Ruiz et al., 2011). Using cell-penetrating peptides that blocked the translocation of AMPARs, it was also shown that preventing AMPAR endocytosis at memory reactivation prevented fear memories from becoming updated (Rao-Ruiz et al., 2011) or sensitive to anisomycin (Hong et al., 2013). Therefore, although activity at AMPARs is more implicated in memory retrieval, there is a correlation between the expression of specific AMPAR subtypes and resistance of established fear memory to modification.

Considering that GluA2-lacking AMPARs are permeable to calcium, it may be that the dynamic of calcium influx is important in supporting memory destabilisation. At least in the hippocampus, another potential neurochemical mechanism underlying memory destabilisation depends upon L-type voltage-gated calcium channels (LVGCCs), blockade of which prevents the destabilisation of reactivated fear memories (Suzuki et al., 2008; Flavell et al., 2011). Additionally, the administration of nefiracetam, a pharmacological agent that enhances LVGCC calcium currents (Yoshii and Watabe, 1994), was shown to enhance the destabilisation of fear memory (Flavell and Lee, 2019).

As noted above, memory destabilisation is induced when expectations are violated at reactivation or, more formally, a “prediction error” is induced. Reward prediction error has been strongly associated with dopamine release from the ventral tegmental area (Schultz et al., 1997) and it also appears that dopamine is required for the destabilisation of both appetitive (Merlo et al., 2015) and aversive memories (Flavell and Lee, 2019). However, dopaminergic signalling does not appear to be *sufficient* to induce memory destabilisation, as enhancing dopaminergic signalling with the D_1_ dopamine receptor agonist SKF38393 did not induce destabilisation of a strong fear memory under reactivation conditions that would normally destabilise a weaker memory (Flavell and Lee, 2019). This may indicate that it is the specific timing of phasic dopaminergic signalling that is critical for memory destabilisation, rather than tonic increases in dopamine.

Intracellularly, memory destabilisation is dependent upon multiple molecular pathways including proteasome signalling and protein degradation. Memory destabilisation depends upon the ubiquitin- and proteasome-dependent degradation of pre-existing postsynaptic proteins, as shown for fear (Lee et al., 2008; Fukushima et al., 2014; Fustiñana et al., 2014; Jarome et al., 2015; Orsi et al., 2019; Tay et al., 2019), spatial (Artinian et al., 2008), object recognition (Furini et al., 2015; Stiver et al., 2017) and drug memories (Ren et al., 2013). It is hypothesised that this mechanism is responsible for the reorganisation of original memory through the degradation of pre-existing synapses and concurrent formation of updated synapses in conjunction with new information presented at retrieval (Kaang et al., 2009; Jarome and Helmstetter, 2013). The regulation of this system has been linked to intracellular calcium signalling (Da Silva et al., 2013; Jarome et al., 2016) and NMDAR activation (Rosenberg et al., 2016).

Despite the advances made in understanding the neurochemical and molecular underpinnings of memory destabilisation, there remains a lack of direct, real-time measurement that destabilisation has occurred. This gap is important not only to provide insight into apparent failures to replicate reconsolidation interference effects, but also for translational studies targeting maladaptive memories in patients, since the learning history and age of the maladaptive memory formation is unique for each patient. Identification of the right parameters to engage destabilisation and avoid boundary conditions, through isolation of a specific and unambiguous biological marker, would constitute a breakthrough in our capacity to modify maladaptive content of naturalistic memories in patients.

## Memory Inhibition Through Extinction

### Pavlovian Extinction and Behaviour

In contrast to the engagement of pavlovian memory reconsolidation by brief exposure to the CS alone, prolonged exposure, or a large number of repetitions of the CS reduces conditioned responding through the process of extinction. During extinction, individuals learn that the CS no longer predicts the emotionally relevant US. It is widely accepted that extinction does not erase or modify the original CS-US memory but inhibits its behavioural expression by establishing a new inhibitory memory trace associating the CS with the absence of the US (CS-noUS; Pavlov, 1927; Bouton, 2004). In clinical settings, pavlovian extinction is the basis of cue exposure therapy, which is widely used for the treatment of specific phobias and PTSD (Rothbaum and Schwartz, 2002).

In common with memory reconsolidation, extinction occurs when the individual experiences prediction error, but requires much longer CS alone exposure. However, memory extinction appears more temporally and spatially context-dependent than memory reconsolidation. The inference that the original CS-US memory remains intact after extinction is based on the findings that behavioural responding to the CS recovers, over time (*spontaneous recovery*), when the extinction context is changed (*renewal*), or after the unexpected presence of the US (*reinstatement*). Also, the residual associative value of the CS allows it to elicit behavioural responses more rapidly than a novel CS (*rapid reacquisition*; Bouton, 2014). However, despite the common requirement for prediction error, reconsolidation and extinction are mutually exclusive processes. Due to the partial overlap in the mechanisms of these processes, the same experimental manipulation can produce bidirectional effects depending on whether reconsolidation or extinction is engaged during re-exposure (Lee et al., 2006a), and the two processes are separated by a “limbo” period during which neither process is engaged (Merlo et al., 2014, 2018; Cassini et al., 2017).

### Neural Mechanisms of Pavlovian Extinction

Reconsolidation and extinction are complex psychobiological processes that require changes in neuronal connectivity supported by neural and molecular events. Even though these processes are supported by distinct brain networks, for pavlovian memories the basolateral amygdala (BLA) is critical for both (Maren and Quirk, 2004; Nader, 2015). Moreover, reconsolidation and extinction engage partially overlapping molecular mechanisms, similar to the relationship between reconsolidation and the initial consolidation of memories. As extinction relies on the formation of a new inhibitory memory, many mechanisms supporting extinction acquisition and consolidation have common and distinctive partners compared to the consolidation of the CS-US memory (for a review see Pagani and Merlo, 2019). Among other mechanisms, extinction consolidation relies on *de novo* protein and mRNA synthesis (Pedreira and Maldonado, 2003), believed to support the synaptic changes necessary for behavioural inhibition.

#### Shared Mechanisms Engaged by Memory Reconsolidation and Extinction

Reconsolidation and extinction are both initiated by similar intracellular and extracellular events, such as activation of NMDARs (Lee et al., 2006a; Flavell and Lee, 2013; Merlo et al., 2014, 2018) and activation of protein kinases (Merlo et al., 2014, 2018). It is well-established that the consolidation of extinction memories depends on NMDAR-mediated changes in synaptic plasticity (Baker and Azorlosa, 1996; Lee et al., 2006a). Santini et al. (2001) have further suggested that consolidation of extinction learning involves a transfer from NMDAR-independent early plasticity to NMDAR-dependent stabilisation that requires protein synthesis. There is also some evidence that the acquisition of the extinction memory, but not its consolidation, is blocked by the administration of GluN2B-selective NMDAR antagonists given shortly before extinction training (Dalton et al., 2012) though not 1 h before training (Cahill et al., 2019). This finding highlights the distinct nature of neural and molecular mechanisms underlying acquisition, consolidation, and retrieval of extinction memory (Santini et al., 2001). Similarly, administration of the NMDAR partial agonist D-cycloserine (DCS) enhances the acquisition of the extinction memory, with no effect when administered after re-exposure in rodents (Ledgerwood et al., 2003; Lee et al., 2006a) and, indeed in human patients undergoing prolonged exposure therapy for phobia (Smits et al., 2013).

As for memory destabilisation, dopamine has also been implicated in pavlovian extinction learning (for review see McNally et al., 2011). Administration of the selective D_1_-dopamine receptor antagonist SCH23390 directly into the BLA impairs the acquisition of extinction, though not its consolidation. By contrast, infusions of SCH23390 into the infralimbic cortex impair extinction consolidation, but not acquisition (Hikind and Maroun, 2008). Furthermore, it has been demonstrated that dopamine receptor antagonism impairs both extinction acquisition as well as the consolidation of extinction memory. Furthermore, the administration of the dopamine precursor L-DOPA enhances the consolidation of extinction in both mice and humans (Haaker et al., 2013). It has also been shown that administration of the D_2_-dopamine receptor antagonist, haloperidol, in the nucleus accumbens (NAc) impaired suppression of fear responses after extinction (Holtzman-Assif et al., 2010). This evidence supports the role of dopaminergic activity in extinction learning and especially pointing towards the NAc as a critical locus for learning and retention of the inhibition created by fear extinction training (Holtzman-Assif et al., 2010). Previous studies investigating extinction have focused mostly on interactions between the BLA and mPFC, however, it has been proposed that dopamine release in the NAc may regulate the interactions between BLA and mPFC (Laurent and Westbrook, 2008) that are required for inhibitory learning during extinction (Holtzman-Assif et al., 2010).

Similar to memory destabilisation, extinction appears to depend upon activation of LVGCCs and protein degradation. Administration of the LVGCC antagonists nifedipine or nimodipine blocked extinction (Cain et al., 2002) with prolonged re-exposure, and blocked the destabilisation of the original memory with brief re-exposure (Suzuki et al., 2004, 2008). Similarly, inhibiting protein degradation by infusing the proteasome inhibitor lactacystin immediately following extinction training prevents extinction of cocaine reward memory in the conditioned place preference procedure, while inhibition of protein degradation following brief exposure prevents memory destabilisation (Ren et al., 2013).

Where molecular mechanisms are common between reconsolidation and extinction, this leads to the possibility that pharmacological interventions could have opposite effects on the pavlovian memory depending upon the extent of CS re-exposure. Considering that maladaptive memories across patients with anxiety disorders or addiction vary in strength and age, the effect of retrieval may vary between patients, with a session inducing memory destabilisation in one case and extinction in another. Thus, the administration of a pharmacological agent to either disrupt the original memory or enhance extinction may worsen symptoms if the cue exposure session engages the alternative memory process (Price et al., 2009; Gerlicher et al., 2019). Targeting molecules or pathways that are differentially engaged by memory reconsolidation or extinction could reduce the risk of manipulating the undesired memory process.

#### Distinct Mechanisms Engaged by Memory Reconsolidation and Extinction

Despite some of the molecular and neural similarities between extinction and reconsolidation mentioned above, marked distinctions are differentiating between these processes at the molecular level.

A clear difference between reconsolidation and extinction mechanisms exists at the level of transcription factors. For example, while NF-κB is necessary for the reconsolidation of fear memory, it is inhibited during extinction training (Merlo and Romano, 2008). By contrast, the nuclear factor of activated T-cells (NFAT) is required for fear extinction, but not for reconsolidation (de la Fuente et al., 2011). A comparable double dissociation has been found with brain-derived neurotrophic factor (BDNF) and the transcription factor Zif268. While both the consolidation of new memory and extinction requires BDNF (Lee et al., 2004; Peters et al., 2010), it is not required for memory reconsolidation, at least for hippocampal-dependent fear memories (Lee et al., 2004). On the other hand, Zif268 *de novo* expression is required for reconsolidation but constrains memory extinction (Lee et al., 2004; Kirtley and Thomas, 2010).

#### Extinction May Involve Some Synaptic Restructuring Events in Common With Memory Destabilisation

The notion that pavlovian extinction involves “unlearning” of the original CS-US memory is difficult to reconcile with the widely reported phenomena of spontaneous recovery, renewal, and reinstatement, all of which indicate that the original memory must remain intact. However, while extinction clearly does involve the formation of a new CS-no US memory that competes with the CS-US memory for behavioural expression, it is possible that the original memory also undergoes some modification, if only to reflect that the CS is now ambiguous, or to incorporate the representation of other cues or contexts that may allow the appropriate response to the CS to be disambiguated (Clem and Schiller, 2016).

Empirically, this view is supported by recent advances in neuronal ensemble research, offering a deeper understanding of how memory engrams can store and retrieve memories. While there do appear to be distinct engrams for fear and extinction memories within the amygdala (Herry et al., 2008), there is also evidence suggesting that extinction learning requires the reactivation of the original memory in both the hippocampus and amygdala (Khalaf et al., 2018; Khalaf and Gräff, 2019).

Recent work indicating that targeting of protein kinases or phosphatases can produce complementary, rather than bidirectional, effects on reconsolidation and extinction further supports the hypothesis that extinctions go beyond the formation of a new CS-noUS inhibitory associative memory to more of a combination of new memory formation and inhibition of original memory (Pagani and Merlo, 2019). While the re-stabilization of a reconsolidating memory depends upon protein kinases (as discussed above), extinction requires the activity of phosphatases and some kinases, and is constrained by kinase activity. The protein kinase CaMKIIα and the protein phosphatase calcineurin have been of particular interest. Phosphoproteomic analyses have revealed that the serine-331 residue on CaMKIIα is differentially regulated by memory reconsolidation and extinction, where inhibitory phosphorylation is decreased and increased respectively (Rich et al., 2016). Furthermore, inhibition of CaMKIIα within the basolateral amygdala disrupted the reconsolidation of a CS-drug memory when combined with brief CS re-exposure and facilitated extinction when combined with prolonged CS re-exposure (Rich et al., 2016). This could provide a promising therapeutic target, as a pharmacological intervention that both disrupts reconsolidation and enhances extinction of maladaptive memories.

CaMKIIα is thought to be a negative regulator of the protein phosphatase calcineurin (Rich and Torregrossa, 2018), which has been extensively studied for its role in extinction. Calcineurin is necessary for the extinction of both contextual (Lin et al., 2003) and auditory fear memory (Merlo et al., 2014). Enhancement of calcineurin activity through the administration of chlorogenic acid both enhances extinction and disrupts the reconsolidation of CS-drug memories, with the effect on extinction being prevented by the co-administration of a calcineurin inhibitor (Rich et al., 2020). The finding that the same molecules—CaMKIIα and calcineurin—can produce opposite effects on both reconsolidation and extinction warrants further investigation.

The hypothesis that extinction involves at least some alteration of the original memory also fits well with layered connectionist (Kehoe, 1988) and statistical models of learning (Dunsmoor et al., 2015; Gershman et al., 2017). The layered connectionist model proposes that pavlovian conditioning does not produce a direct link between the CS and CR, but rather associates in separate layers the CS and CR with an intermediate element, X. According to this view, extinction weakens the CS-X association, while leaving the X-CR association relatively intact. This conceptualisation therefore allows for both unlearning (of CS-X) and preservation (of X-CR) to occur simultaneously. In statistical models of learning, the degree to which the original memory is updated vs. new learning happening depends on inferring whether or not the current trial can be associated to the original latent cause—in which case, the original memory will be updated—or if it is different enough to require new latent cause grouping—in which case a new extinction memory will form. This also means that the memory updating vs. new learning balance is influenced by the training protocol used during extinction training. When the conditions during extinction training are more similar to the original training context, it is more likely that the original memory will be updated or “unlearned.” According to Gershman et al. (2017), updating mechanisms depend on the reconsolidation of the original memory, while new learning is dependent upon the extinction processes.

## The Space in Between

As mentioned above, the behaviourally opposing memory mechanisms of reconsolidation (affecting the original CS-US memory) and extinction (promoting the formation of an inhibitory CS-noUS) are linked by the common environmental event of CS re-exposure. The addition of further unreinforced CS re-exposure, therefore, has a non-linear effect on the individual’s behavior, producing a dramatic effect on the individual’s behavioural repertoire and promoting a variety of cellular and molecular modifications in a myriad of brain regions. Surprisingly, the behavioural and mechanistic properties of the transition between memory states—sometimes referred to as “the null point” or “limbo”—produced by increasing the number of unreinforced CS re-exposure has only recently begun to capture research interest. It has been observed for discrete auditory fear memories in rats, where bidirectional manipulations of NMDAR activity affected memory reconsolidation or extinction when administered before brief or prolonged CS re-exposure sessions respectively (Lee et al., 2006a), but no effect when administered before an intermediate number of CSs (Merlo et al., 2014). Similar results were observed for the pavlovian conditioned approach (Flavell and Lee, 2013) and contextual fear memories in rats (Cassini et al., 2017; Franzen et al., 2019), and fear memories in humans and crabs (Sevenster et al., 2014; Merlo et al., 2019), suggesting that “limbo” is an evolutionarily conserved feature in retrieval-dependent associative memory processing.

As this insensitive or limbo phase is associated with an intermediate number of CS presentations, there are two potential accounts for the apparent lack of effect of pharmacological manipulations. One is that the intermediate number of CS re-exposure fails to engage either memory reconsolidation (due to excessive CS re-exposure) or extinction (due to insufficient CS re-exposure) within a single individual. An alternative at the population level is that intermediate CS re-exposure engages reconsolidation in some animals while engaging extinction in others. According to this second account, the amnestic agent (e.g., MK801) should have a similar deleterious effect on the CR in both the reconsolidating and extinguishing individuals, but as the effect would be to reduce fear at test in the reconsolidating individuals and to enhance fear at test in the extinguishing individuals, these effects would effectively cancel out at the population level. This is mechanistically possible, but somewhat improbable since it implies both processes should be equally sensitive to the manipulation. Detailed analysis of a large number of fear-conditioned rats injected with MK801 before an intermediate duration context exposure showed that there was no reduction in the correlation between freezing levels at re-exposure and test sessions, no differential effects within subpopulations, and no change in variability compared to saline-treated controls (Cassini et al., 2017). Moreover, at the molecular level, limbo is associated with no change in ERK1/2 activation in the BLA, which is increased during both reconsolidation and extinction (Merlo et al., 2018). This suggests that intermediate CS re-exposure engages the distinct mnemonic process of limbo, characterised by the absence of known retrieval-dependent plasticity mechanisms and memory processes.

During limbo, the memory is not only insensitive to NMDAR manipulations. Administration of midazolam, an enhancer of GABAergic activity, also failed to affect contextual fear conditioned responding after intermediate CS re-exposure (Alfei et al., 2015; Franzen et al., 2019). Furthermore, protein synthesis inhibition, a “gold standard” amnestic manipulation, did not affect conditioned responding when administered after an intermediate number of CS re-exposures in the crab *Neohelice granulatus* (Merlo et al., 2019). Altogether, these data support the hypothesis that intermediate CS re-exposure engages limbo, a state where extinction does not take place, but also where the original CS-US memory is insensitive to well-tested amnestic interventions. Limbo may therefore represent the CS “space” where there is too much novel information regarding the CS to allow modification of the existing memory, but not enough to engage new learning. Alternatively, limbo may be a completely new process, with distinctive neural mechanisms, affecting conditioning responding in an unestablished manner. Further investigation is needed to distinguish between these alternatives or propose new hypotheses.

A better understanding of limbo may help to explore further the mutually exclusive nature of memory reconsolidation and extinction, and how they interact to determine memory persistence or inhibition. Pharmacologically manipulating the limbo state, if possible, could be used to extend CS-US memory lability into longer CS re-exposure protocols and thereby delay extinction, increasing the window of opportunity to alter maladaptive naturalistic memories in patients.

## Conclusions and Future Directions

Considering the complex relationship between memory destabilisation/reconsolidation, limbo, and extinction, and their respective boundary conditions (Merlo et al., 2014; Cassini et al., 2017), any potential therapeutic intervention that aims to target one of these processes has the potential to result in unexpected effects on the other process, leading to the possibility of maintaining or even enhancing maladaptive memories (Lee et al., 2006a; Tronson et al., 2006). As already noted, to achieve greater reliability of treatment strategies and insight into the apparent fragility of memory reconsolidation, it is necessary to identify clear markers for memory destabilisation and subsequent memory processes. One promising approach could be event-related potentials, which can distinguish unique neurophysiological markers for consolidated, reconsolidated, or extinguished memories (Mueller et al., 2014; Campos-Arteaga et al., 2020). Applied to humans, such markers would allow the application of more effective treatments for those suffering from maladaptive emotional memories. Preclinically, this type of approach could help to determine whether apparent contradictory findings and failed replications of reconsolidation manipulations are due to a failure to engage memory destabilisation. Moreover, precise characterisation of the exclusive cellular and molecular mechanisms of memory destabilisation and extinction will help to determine the effects of different retrieval-associated manipulations. This insight, mainly to be obtained in animal paradigms, is crucial for evaluating the translational potential of memory modification to decrease maladaptive behavior in clinical practice.

## Author Contributions

AM and EM conceptualised the manuscript. ZV, AM, and EM researched the literature. ZV produced the first draft of the manuscript. AM and EM reviewed and edited the manuscript. All authors contributed to the article and approved the submitted version.

## Conflict of Interest

The authors declare that the research was conducted in the absence of any commercial or financial relationships that could be construed as a potential conflict of interest.
